# Establishment and Evaluation of a Monkey Acute Cerebral Ischemia Model

**DOI:** 10.6061/clinics/2020/e1339

**Published:** 2020-02-18

**Authors:** Li Yan, Xiaodong Zhou, Xiaobin Yang, Yu Zheng, Chunying Liu, Lili Zheng, Ling Fang, Wen Luo, Guangbin He, Jianguo He, Jianmin Zheng, Yin Zhou

**Affiliations:** I Institute of Medical Research, Northwestern Polytechnical University, 127 West Youyi Road, Xi'an, Shaanxi 710072, China; II Department of Ultrasonography, Xijing Hospital, The Fourth Military Medical University, Xi'an 710032, China; III Ultrasound Diagnosis & Treatment Center, Xi'an International Medical Center, Xi'an 710100, China; IV Department of Radiology, Xijing Hospital, The Fourth Military Medical University, Xi'an 710032, China; V Department of Ultrasonography, Xi'an Central Hospital, The Third Affiliated Hospital of JiaoTong University, Xi'an 710003, China; VI Department of Ultrasonography, Xi'an Children’s Hospital, The Affiliated Hospital of JiaoTong University, Xi'an 710003, China

**Keywords:** Cerebral Ischemia, Animal Model, Autologous Thrombus, Middle Cerebral Artery (MCA), Digital Subtraction Angiography (DSA), Magnetic Resonance Imaging (MRI)

## Abstract

**OBJECTIVES:**

Cerebral ischemia seriously threatens human health and is characterized by high rates of incidence, disability and death. Developing an ideal animal model of cerebral ischemia that reflects the human clinical features is critical for pathological studies and clinical research. The goal of this study is to establish a local cerebral ischemia model in rhesus macaque, thereby providing an optimal animal model to study cerebral ischemia.

**METHODS:**

Eight healthy rhesus monkeys were selected for this study. CT scans were performed before the operation to exclude cerebral vascular and intracranial lesions. Under guidance and monitoring with digital subtraction angiography (DSA), a microcatheter was inserted into the M1 segment of the middle cerebral artery (MCA) via the femoral artery. Then, autologous white thrombi were introduced to block blood flow. Immediately following embolization, multisequence MRI was used to monitor cerebrovascular and brain parenchymal conditions. Twenty-four hours after embolization, 2 monkeys were sacrificed and subjected to perfusion, fixation and pathological examination.

**RESULTS:**

The cerebral ischemia model was established in 7 rhesus monkeys; one animal died during intubation. DSA and magnetic resonance angiography (MRA) indicated the presence of an arterial occlusion. MRI showed acute local cerebral ischemia. HE staining revealed infarct lesions formed in the brain tissues, and thrombi were present in the cerebral artery.

**CONCLUSION:**

We established a rhesus macaque model of local cerebral ischemia by autologous thrombus placement. This model has important implications for basic and clinical research on cerebral ischemia. MRI and DSA can evaluate the models to ensure accuracy and effectiveness.

## INTRODUCTION

The cerebral vascular anatomy of non-human primates is highly similar to that of human beings (1). As such, primate models of cerebral ischemic infarction are an important tool to study the pathogenesis, injury mechanisms, diagnosis, prevention and treatment of human cerebrovascular diseases (2-4). At present, common cerebral ischemia models are mostly based on mice, rabbits and dogs, and are generated using ligation, venous injury, transcarotid silk suture embolization, and thromboembolism (5-7). Clinically, cerebral ischemia is mainly caused by thromboembolism (8), with white thrombi being the main component. Approximately 85% of cerebral ischemia cases are caused by thromboembolism in the middle cerebral artery (MCA) (9). Hence, we chose monkeys, which are anatomically similar to humans, to develop an animal model of cerebral embolism. Based on our previous experimentation, we developed a modeling method that involves introducing autologous white thrombi into the MCA; the model was then assessed with imaging analyses and laboratory tests. Our study may provide a suitable animal model to study cerebral ischemia.

## MATERIALS AND METHODS

### Experimental animals

After approval by the Institutional Animal Care and Use Committee of The Fourth Military Medical University, eight male macaques (8-10 kg, mean: 8.99±0.63 kg; 7-8 years, mean: 7.5±0.43 years) were purchased from Fangchenggang Changchun Biotechnology Development Co., Ltd., Guangxi (Approval No., SCXK Gui 2013-0004).

### Experimental methods

#### Preparation of autologous thrombosis

Approximately 2 mL of femoral arterial blood was drawn from each monkey and immediately centrifuged at 4,000 r/min for 10 min. One milliliter of supernatant was collected, mixed with 100 U of thrombin and transferred into a silicone tube with a diameter of 1.5 mm. The specimens were then kept at 4°C for subsequent analysis to determine the size of thrombi to be injected during the operation.

#### Modeling process

Prior to operation, each monkey was fasted for 8h, including a 4h water fast at room temperature. The animals were then anaesthetized via abdominal injection with 3% pentobarbital sodium at a dose of 1 mL/kg. To maintain an adequate level of anesthesia, half of the initial dose was administered every 2h during the experiment. After successful anesthesia, preoperative CT scans were performed to exclude intracranial lesions.

Each animal was immobilized on the operating table in the supine position. The skin in the vicinity of the lateral femoral vein area of the left leg was prepared, disinfected, and punctured. Subsequently, the femoral vein was connected to a three-way tube for blood collection and drug administration. The animal was then provided with a nasogastric feeding tube and oxygen supply. We continuously monitored routine ECG, respiration, blood pressure and anesthetic status.

The right femoral artery was selected as the puncture point, and local anesthesia was delivered with 1% lidocaine after routine skin preparation and disinfection. The right femoral artery was dissociated, and a 4F catheter (Renegade Hi Flo, USA) was inserted through the 4F femoral artery sheath. Under the guidance of DSA fluoroscopy (Siemens, AXIOM Artis dBA, Germany), the catheter was inserted into the right common carotid artery to the proximal end. Anterior and lateral angiography was performed to determine the flow in blood vessels. An injected contrast agent (total volume 1.8 mL, velocity 0.8 mL/s) also confirmed that the catheter was in the right internal carotid. Through this catheter, a 1.9 F microcatheter (Johnson & Johnson, Prowler, USA) was introduced into the M1 segment of the cerebral artery, as detected by the contrast agent. One or two thrombi were drawn into a 1 mL syringe and injected through the microcatheter to the distal end; the thrombi were not revealed by the contrast agent. Anterior and lateral angiography was performed again to determine successful embolization before the microcatheter was withdrawn. After vascular occlusion and ischemia were confirmed, antibiotics were administered intravenously to prevent infection. Following embolization, multisequence MRI (GE Discovery MR750, USA) was used to monitor cerebrovascular and brain parenchymal conditions (3D-TOF-MRA: ET=1, TR/TE: 15.1 ms/4.0 ms, DFOV: 6.6×6.6 cm; Propeller FSE T2WI: ET=32, TR/TE: 4636.7 ms/96.2 ms, DFOV: 20.0×20.0 cm; SE T1WI: ET=10, TR/TE: 1850.0 ms/21.8 ms, DFOV: 20.0×20.0 cm; SE EPI: ET=1, TR/ TE: 3000.0 ms/72.2 ms, DFOV: 20.0×20.0 cm).

#### Specimen acquisition and observations

Twenty-four hours after generating the models, 2 monkeys were sacrificed and fixed in 10% formalin solution after systemic perfusion. The fixed heads were left intact. Two weeks later, craniotomies were performed to harvest the brain tissue. Coronal sections approximately 5 mm in thickness were generated and prepared for analysis as follows: routine fixation, dehydration, clearing, paraffin embedding, sectioning, and hematoxylin-eosin (HE) staining; the sections were observed by light microscopy.

## RESULTS

### General results of modeling

The model was successfully established in 7 monkeys. One animal died of intracerebral hemorrhage during intubation because the MCA of the monkey has a smaller lumen diameter and is more tortuous than that in humans. In addition, the surgeon was relatively inexperienced during the first catheterization attempt using a larger microcatheter, which punctured the vascular wall during advancement into the MCA. In the other cases, through the interventional operation, autologous white thrombi were introduced into the MCA to facilitate embolization. We observed the thrombi and the presence of the infarcted areas. These observations were verified by DSA, MRI and post-operative pathological examination.

### Imaging manifestations

#### DSA assessment

**Figure 1:**
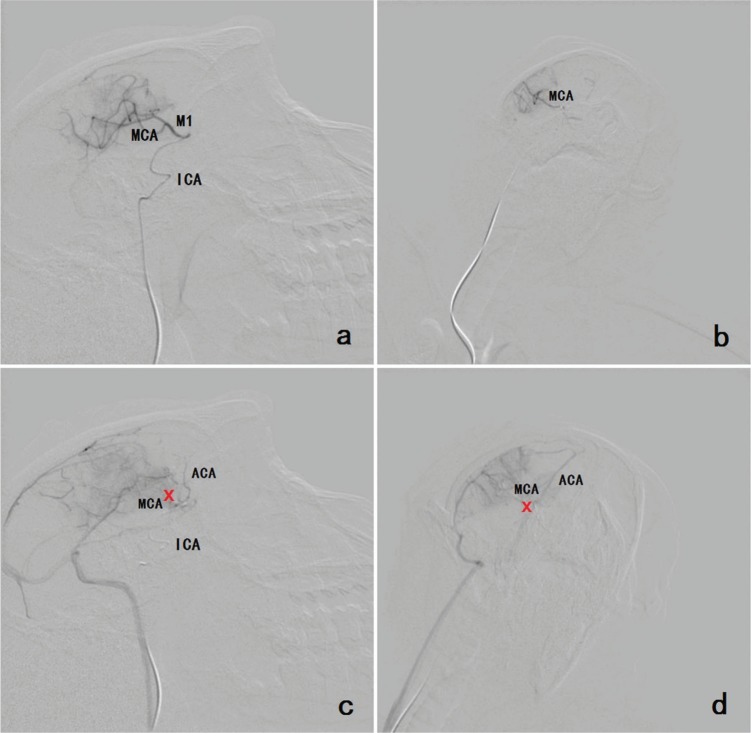
**a, b** Before the operation, DSA revealed the MCA and its segments and the ICA (a: lateral position; b: anteroposterior position). **c, d** After the operation, DSA showed that the MCA and its segments were occluded. The reflux of the contrast medium caused enhancement of the ACA. The occlusion is indicated by the red cross (c: lateral position; d: anteroposterior position). Footnote: (MCA=middle cerebral artery; ACA=anterior cerebral artery; ICA=internal carotid artery).

Before the operation, DSA revealed the MCA and its segments. During the operation, DSA facilitated real-time, dynamic monitoring. After the operation, DSA showed that the MCA and its segments were occluded (Figure 1).

#### MRI assessment

**Figure 2:**
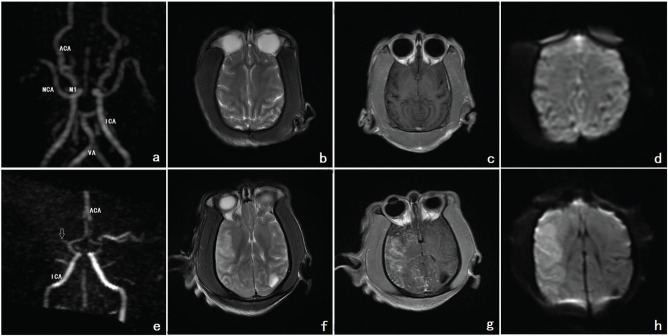
**a, b, c, d** Before the operation, MRA showed the MCA and its segments. The ACA, ICA and VA can also be seen. On T2, contrast-enhanced T1 and DWI imaging, the normal cerebral parenchyma of the MCA showed iso-signal intensity. **e, f, g, h** After the operation, MRA observed that the MCA and its segments were occluded. The occlusion is indicated by the white arrow. On T2, contrast-enhanced T1 and DWI imaging, the MCA demonstrated high signal intensity. Cerebral infarction formation in the parenchymal area was revealed. Footnote: MRA refers to images a and e; T2 refers to images b and f, contrast-enhanced T1 refers to images c and g; DWI refers to images d and h (MRA=magnetic resonance angiography; MCA=middle cerebral artery; ACA=anterior cerebral artery; ICA=internal carotid artery; VA=vertebral artery; DWI= diffusion-weighted imaging).

Before the operation, MRA detected the MCA and its segments. T2, contrast-enhanced T1 and diffusion-weighted imaging (DWI) images showed iso-signal intensity of the normal cerebral parenchyma of the MCA. After the operation, MRA indicated that the MCA and its segments were occluded. T2, contrast-enhanced T1 and DWI imaging demonstrated high signal intensity of the MCA region. A cerebral infarction in the parenchymal area was revealed (Figure 2).

### Pathological observation

#### Observation of pathological gross specimens

**Figure 3:**
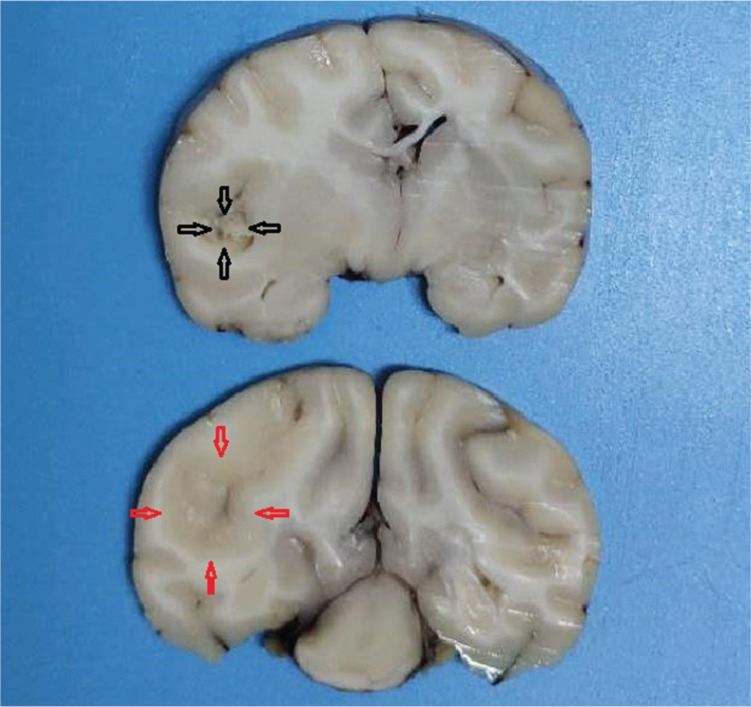
Two sections of the specimen were observed. The infarct lesion was fainter and softer than the surrounding normal tissue; necrosis was detected in the center. The red arrow shows the boundary, while the black arrow shows the necrosis of the infarct lesion.

After craniotomy, the infarct lesions were fainter and softer than the surrounding normal tissue; necrosis was detected in some infarct areas (Figure 3).

#### HE staining of the lesions

**Figure 4:**
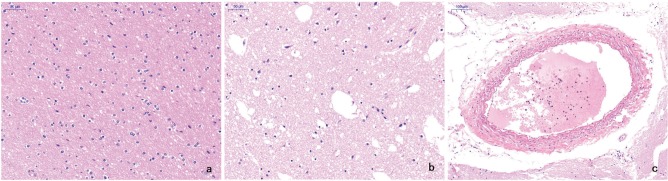
**a** In normal brain tissue, both the gray matter and white matter contained normal glial cells and neuronal structures (HE, original magnification ×50). **b** The infarct area contained sparse nerve fibers, nerve cells with different degrees of ischemic alterations, fewer glial cells, and an overall mesh-like pathological alteration. The relatively increased phagocytic microglia also indirectly reflected necrosis (HE, original magnification ×50). **c** The arterial wall was thickened and intravascular thrombi were visible in the blood vessels in the infarct areas (HE, original magnification ×100).

In normal brain tissue, both the gray matter and white matter contained normal glial cells and neuronal structures. In contrast, the infarct areas contained sparse nerve fibers, nerve cells with different degrees of ischemic alterations, fewer glial cells, a relative increase in phagocytic microglia, and an overall mesh-like pathological alteration. The arterial wall was thickened, and intravascular thrombi were visible in some blood vessels in the infarct areas (Figure 4).

## DISCUSSION

In this study, we performed DSA-guided vascular intervention to introduce autologous white thrombi into the M1 segment of the MCA in 8 monkeys, thereby establishing an animal model of cerebral ischemia. One of the monkeys died during catheterization because the vascular lumen in rhesus macaques is narrower than that in humans; in particular, the MCA has a small lumen diameter and is tortuous. During the first catheterization attempt, the surgeon was relatively inexperienced and used a large microcatheter, which punctured the vascular wall during advancement into the MCA. This resulted in intracerebral hemorrhage and death during the operation. For the other 7 monkeys, the model was successfully established, as determined by MRI and DSA. This model, which uses interventional intubation of autologous thrombi, has the following advantages: i) minimal surgical trauma and complications; ii) intuitive intubation and embolization with accurate positioning; iii) stable area of infarction formation that is predominantly in the blood supply area of the MCA; iv) similarity of the autologous white thrombi to the common thrombus components of the cerebral embolism and consistency with human pathogenesis; and v) flexible and reliable model evaluation with dual MRI and DSA imaging.

The study of animal models for cerebrovascular diseases can improve our understanding of disease mechanisms, identify candidate genes for related human disorders, and provide experimental models for preclinical trials (7,10). Progress in understanding stroke is dependent on animal models of focal cerebral ischemia (11). However, the method by which a reliable cerebral infarction animal model could be established remains a great challenge for medical research. Non-primates are relatively small, the nervous system is underdeveloped, and the human brain structure is quite different; thus, they are often employed as initial research subjects. In comparison with conventional animals, nonhuman primate models offer distinct advantages including similar human collateral circulation, cerebral hemispheric blood flow, and perfusion ratios of white matter to gray matter (12-15). Hence, rhesus macaque was selected for our study because of its similarity to the human cerebrovascular system (1,4).

The MCA, which is the most common site of ischemic stroke in humans, does not have abundant collateral circulation. Hence, it is generally accepted that middle cerebral artery occlusion (MCAO) is the standard method to develop animal models of focal cerebral ischemia. Traditional methods for generating MCAO include ligation, vein injury, transcarotid silk suture embolization, craniotomy and electrocoagulation, photochemistry, and nonthrombus-based embolization (5,6,16-18). These methods often lead to considerable trauma, high mortality, and incorrect embolism sites. More importantly, these methods are not consistent with the actual disease traits because most clinical MCAO cases are caused by thromboembolism (19,20). To prepare thrombi, some researchers use autologous venous or arterial blood to produce red thrombi and embolization after their coagulation. In this study, we found that this type of thrombus easily autolyzed and led to spontaneous recanalization. In addition, human ischemic cerebral infarction is caused by embolization of white thrombi originating from atherosclerotic plaque shedding. Hence, we were inspired by Wei and Lin’s method (21) to produce autologous white thrombi, which are mainly composed of platelets and are less likely to autolyze. Based on the vascular diameter detected with imaging, each thrombus was microscopically divided into emboli of an appropriate size. This enabled emboli measurement and, in turn, increased the success rate of embolization.

In this study, MRI and DSA imaging methods were used to evaluate the craniocerebral vessels in animals before and after the operation. Our results showed that MRI not only allowed multiplane imaging of cerebral vessels but also produced real-time assessment of the brain parenchyma. In addition, DSA facilitated and evaluated the interventional procedure dynamically, intuitively, and in real-time. The dual assessment with MRI and DSA ensures the effectiveness and accuracy of the operation (22).

In this study, an interventional approach was employed to establish a cerebral infarction model. This approach featured minimal trauma and a high success rate. The evaluation criteria were straight-forward and objective. However, due to the scarcity and high cost of the animals, the interventional operation had stringent requirements and expensive equipment and medicine. Consequently, the sample size was small, and it is currently difficult to widely apply this approach. Nonetheless, this primate model of cerebral ischemia generated by autologous white thromboembolism in the MCA is very similar to the actual disease. In the future, we will use this model to carry out basic research and clinical trials on thrombolytic therapy (e.g., pathogenesis and the timing, administration, efficacy, and safety of therapies) (23). Moreover, cerebral vessel imaging with super-resolution will also be incorporated to evaluate thrombolysis (24). In other words, to provide a more reliable and effective experimental platform to investigate the occurrence and development of stroke diseases, a multimodal image evaluation system will be developed to monitor the model and thrombolytic outcomes to further optimize model generation and integrate the imaging data.

## AUTHOR CONTRIBUTIONS

Zhou X is the guarantor of the integrity of the entire study. Zhou X and Yan L were responsible for the study concept and design. Yang X was responsible for the literature research. Yan L and Yang X were responsible for the clinical studies. Zheng Y and Fang L were responsible for the experimental studies and data analysis. Luo W and He G were responsible for the quality control of the data. He J and Zhen J were responsible for the manuscript preparation. Zhou Y was responsible for the manuscript editing. Liu C and Zheng L were responsible for the manuscript revision.
